# Machine Learning and Systems Biology Approaches to Characterize Dosage-Based Gene Dependencies in Cancer Cells

**Published:** 2021-02-26

**Authors:** Kevin Meng-Lin, Choong Yong Ung, Taylor M Weiskittel, Alex Chen, Cheng Zhang, Cristina Correia, Hu Li

**Affiliations:** 1 Center for Individualized Medicine, Department of Molecular Pharmacology and Experimental Therapeutics, Mayo Clinic College of Medicine, Rochester, MN, USA; 2 Information Systems and Robotics at Carnegie Mellon University, Pittsburgh, Pennsylvania, USA

**Keywords:** Gene dependencies, Machine learning

## Abstract

Mapping of cancer survivability factors allows for the identification of novel biological insights for drug targeting. Using genomic editing techniques, gene dependencies can be extracted in a high-throughput and quantitative manner. Dependencies have been predicted using machine learning techniques on –omics data, but the biological consequences of dependency predictor pairs has not been explored. In this work we devised a framework to explore gene dependency using an ensemble of machine learning methods, and our learned models captured meaningful biological information beyond just gene dependency prediction. We show that dosage-based dependent predictors (DDPs) primarily belonged to transcriptional regulation ontologies. We also found that anti-sense RNAs and long- noncoding RNA transcripts display DDPs. Network analyses revealed that SOX10, HLA-J, and ZEB2 act as a triad of network hubs in the dependent-predictor network. Collectively, we demonstrate the powerful combination of machine learning and systems biology approach can illuminate new insights in understanding gene dependency and guide novel targeting avenues.

## 1. Introduction

In the era of targeted therapy, understanding cancer fitness is critically important for future therapeutic development. Genes hinge on each other to define context specific phenotypes and our understanding of such interactions is paramount for better drug targeting. The term gene dependency was coined to describe genes that a particular cancer cell cannot proliferate without. Genetic screens have been used to identify gene dependencies [1]. Tsherniak et al. performed one of the most inclusive screens using RNAi in human cancer cell lines [2]. They then constructed regression conditional inference trees to model a given gene’s dependency based on somatic mutations, gene copy number, and gene expression. Surprisingly, 82% of predictable differential dependencies were best predicted by gene expression alone. This finding suggests that gene dependency is primarily dictated by transcriptomic features. Chen et al. added to this work by mapping gene dependency at the proteomic level [3]. Broadly, proteomics is considered less practical for systems analysis due to its variation across protocols, scarcity in public repositories, and lower gene coverage. Despite these challenges, Chen et al. revealed a similarly strong predictive power of proteomics in gene dependency. While both groups linked gene dependencies to other genes predictive of their function, neither explored the biological associations between dependency predictor gene pairs. Thus, the biological significance of gene dependency and predictor relationships has been largely unexplored. We hypothesize that the relationships between a gene dependency and its predictors can be captured at the transcriptomic level and encompass valuable information on gene dependency biological mechanisms and cancer fitness. Specifically, we examined the transcriptomic landscape for a specific type of predictor called dose-dependent predictors (DDPs). Dose-dependent predictors are predictors that show dose response behavior with gene dependency. Here, we developed a machine learning procedure termed Expression Dosage Dependent Inferelator (EDDI) that can enable the identification of predictive dependency genes and elucidate their biological relationship in human cancer cell lines. In this way, we leveraged the learned features from the machine learning model in systems biology approaches to further biological understanding.

## 2. Method

### 2.1 Data

Gene expression and dependency data was obtained from the depmap portal (https://depmap.org/portal/) [2]. CCLE RPKM data from release 18Q3 was used for this study. This dataset included 487 cancer cell lines spanning 21 tissue and 32846 genes [4, 5]. DEMETER inferred dependency scores for 17098 genes across 501 cell lines were obtained from Achilles release 2.20.2 [6]. Expression data was transformed to log2(RPKM+1). Hematopoietic cell lines were removed from the datasets due to their unique genetic background compared to solid tissue tumors, and common cell lines between the two datasets were kept, leaving 440 cell lines spanning 20 tissues. We refer to dependencies as genes from the set of 17,098 with dependency scores.

### 2.2 Initial Screen

**Figure 1:**
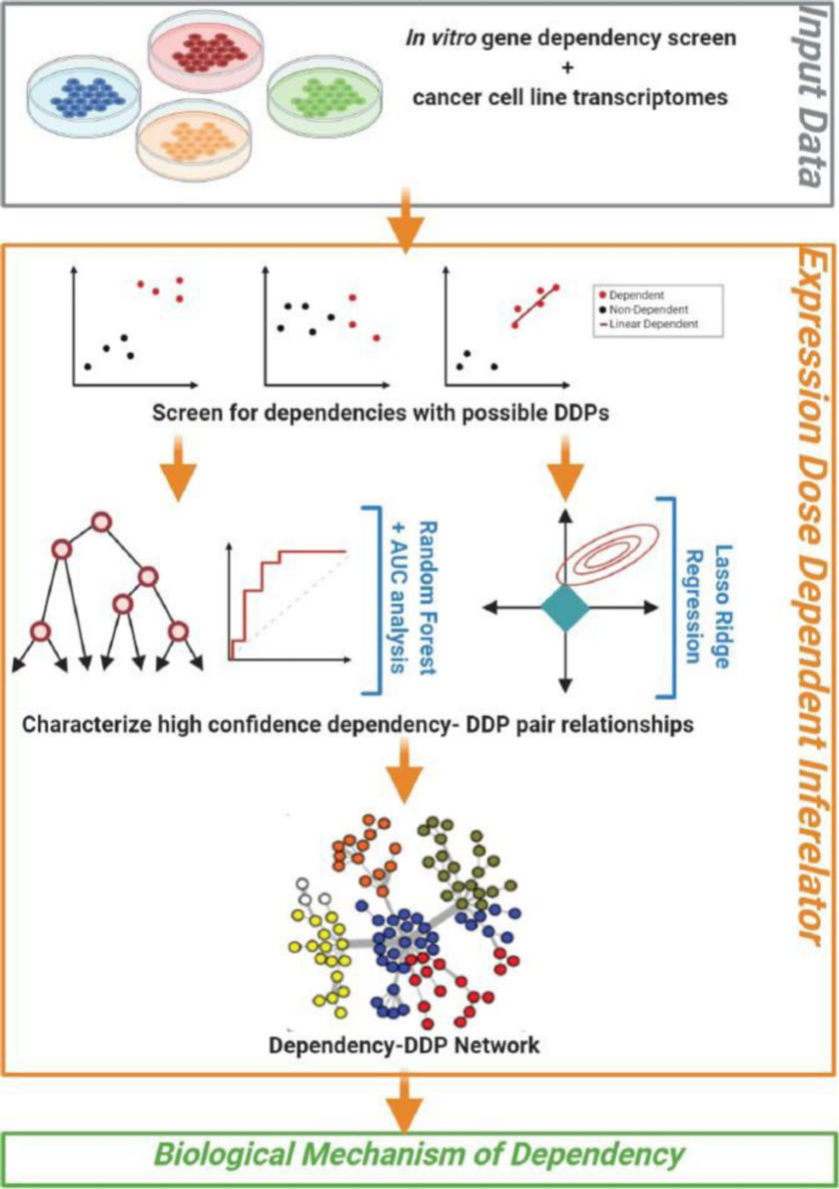
Schematic illustration of Expression Dose Dependent Inferelator (EDDI). Using dependency screening and RNAseq data, our study uses machine learning methods to discriminate modes of dependency and allow for the identification of dosage-based dependency predictor genes (DDPs). DDPs gene expression patterns in dependent and non-dependent cancer cells were used to predict dependency to a given gene. Ensemble models corresponding to each gene dependency were constructed and evaluated. The resulting dependent-predictor pairs obtained across all trained models captured dosage-based dependencies which were used to construct networks for dependent and predictor genes. Our proposed methodology allows us to extract biological mechanisms of dependency.

Pairwise screening was performed for all combinations of dependencies and genes to identify genes showing expression-based separation between dependent and non-dependent cell lines, as well as linear expression in the dependent cell lines (Figure 1). Due to the lack of a strict dependency score threshold denoting dependency, different thresholds were tested for each pair to find a naïve boundary and assess predictive viability. For a given dependency D (range of - to −2) cell lines with dependency scores less than 0 were used as an initial set of “dependent” cell lines and the remaining cell lines were the initial set of ‘non-dependent’ cell lines. For a given gene G, a 10-fold cross-validated logistic regression model was fitted using G’s expression a predictor and D’s labels. The brier score which is used for binary outcomes, was calculated for each fold and averaged to give the final cross- validation brier score [7]. This process was repeated iteratively by dropping the highest scoring cell line from the dependent set until only 20 cell lines remained. The dependency score associated with the lowest cross-validated brier score was chosen as a tentative boundary to classify ‘dependent’ and ‘non-dependent’ cell lines. Pairs were then filtered based on whether the dependent cell lines’ expression 75^th^ percentile of G was greater than or equal to that of the non-dependent set’s expression 25^th^ percentile of G and *vice versa*. This step was performed to determine whether there may be good expression-based separation between the dependent and non-dependent cell lines. To screen for potential linearity in the remaining pairs, a relaxed lasso model was fit on the dependent set of cell lines using G’s expression as a predictor and D’s scores as the labels. Only genes whose linear models had a significant slope (P<0.001) were kept as a potential predictive feature for their respective D.

### 2.3 Defining Dependent Cell Lines

The previous dependent/non-dependent score boundaries were designed solely to identify dependency-gene pairs where expression may control the dependency. A strict dependency classification was needed to proceed with analysis. We initially set dependency score threshold of −2. However, a threshold at −2 may not be enough to reveal significant differences between the two dependency classes. To remedy this, each dependency (D) and expression (G) pair passing the previous filtering step was clustered using k-means clustering with k=2 and a minimum cluster size of 20 cell lines. The cluster with the most cell lines below −2 was deemed the ‘dependent’ cluster, and cell lines with dependency scores < −2 in this cluster were labeled as dependent. For a given dependency D, the union of all dependent cell lines from the pairwise clustering became the final set of dependent cell lines.

### 2.4 Classification Models

#### 2.4.1 Random Forest:

A random forest classification model was built for all dependencies. The previously determined gene sets for each dependency were used as features for their respective models. Models were constructed using a constant size of 500 trees and minimum node size of 1. The number of predictors to consider at each split (m) was tuned using the randomForest package’s tuneRF function [8]. We assessed our model with a stratified 10-fold cross validation scheme repeated 5 times using the AUC as our performance metric (roc package) [9]. To account for the lack of dependent cell lines in comparison to non-dependent lines, dependent cell lines were up-sampled in each fold. We collected the variable importance for each model using the randomForest package’s varImpPlot function, which reports the mean Gini index decrease for each feature.

#### 2.4.2 Regression Modeling:

Dependencies which had a final cross-validated AUC of 0.7 or higher were considered for lasso regression modeling. Regression models were built for the dependent cell lines only. Using the feature set determined from the initial screen, we first normalized the expression data using a z-score transformation. We then used the glmnet package [9] to fit a relaxed lasso regression model to our data and selected the optimal hyper parameters γ and λ using leave-one-out cross-validation [10].

### 2.5 Characterizing Gene-Dependency Relationships

Predictive genes from the well performing random forest models were grouped into broad classes that describe their expressions patterns between dependent and non-dependent cell lines. For each of the dependencies whose random forest classifiers achieved and AUC>0.7, we computed their differentially expressed gene sets between dependent and non-dependent cell lines using DESeq2 [11]. We compared the results to the respective model’s feature list. Predictive genes in the random forest model with at least a 2 fold expression difference and an adjusted p- value<0.05 were identified as over or under-expressed genes depending on the direction of the expression difference. Predictive genes demonstrating extreme under-expression in dependent cell lines with a log2(RPKM+1) expression less than 0.1 were labeled as “suppressed under-expressed” genes. In addition to differential expression analysis, we also used a template matching scheme where a binary vector was constructed for each dependency [12]. Dependent cell lines were represented as “1” and non-dependent cell lines were represented as “0” in this binary template vector. The Pearson correlation coefficient between each template and its corresponding predictive genes was computed. Genes with a correlation coefficient magnitude of at least 0.5 were given the “separable” label to denote the distinct expression patterns between dependent and non-dependent sets. Lastly, predictive genes from the relaxed lasso models were given the “SEESAW” label to indicate a linear scaling of this gene’s expression on the dependency score of the gene’s corresponding dependency. The list of well performing dependencies, their predictive genes, and the relationship between predictor and dependency is provided in Data S1.

### 2.6 Pathway enrichment analysis for dosage-based dependency predictor genes

Pathway enrichment analyses for dosage-based dependency predictor genes (DDPs) for 16 gene dependencies were performed using WebGestalt (http://www.webgestalt.org/) on KEGG pathways against the human genome [13]. Pathways with at least 5 genes overlapped with query pathway datasets and FDR<0.25 computed with Benjamini- Hochberg (BH) multiple adjustment test were deemed enriched.

### 2.7 Code availability

Codes for this work can be accessed freely at https://github.com/HuLiLab/EDDI for academic use.

## 3. Results

### 3.1 Definition and search of dosage-based dependency predictor genes (DDPs)

Gene expression patterns seen in cancer cells reflect upstream signaling events, genomic mutations, and epigenetic variations. Typically, the expression of a given gene is affected by the expression of many other genes and as such genome-wide expression profiles are usually nonlinear. Gene expression and gene dependency are also highly context specific, and thus we hypothesized that DDPs (dose-dependent predictors) are specific to cancer type. Here, we used CCLE compendium to identify DDPs and we defined three major types of dosage-based gene expression patterns: over-expression, under-expression, and “SEESAW”-like expression. The expression patterns for over- and under-expression were non-linear. In the under- and over-expression categories, a subcategory of genes clearly separated dependent from non-dependent cell lines and thus were termed separable (Figure S1–S3). Furthermore, in the under-expression category certain gene’s expression was almost completely suppressed so we delineated this as a third more extreme category of suppressed under-expression. A third category of SEESAW expression was specified for genes that had linear expression patterns for dependent cell lines. SEE described the linear increase of a gene in dependent lines, and SAW described the linear decrease. Examples of these expression patterns are shown in Figure S1–S3. With these expression patterns defined, we performed a systematic search for candidate dosage-based dependency predictor genes (DDPs). We searched across 440 human cancer cell lines derived from 21 tissue types each with 17,098 gene dependency scores generated by Tsherniak et al. and corresponding CCLE expression data. Using an unbiased approach, we devised a novel strategy to exhaustively search for thresholds that best characterize a dependency (see Methods). Our approach contrasts with others that used a hard cut-off score to investigate gene dependency.

### 3.2 Dosage-based dependency predictors provide biological insights

**Figure 2:**
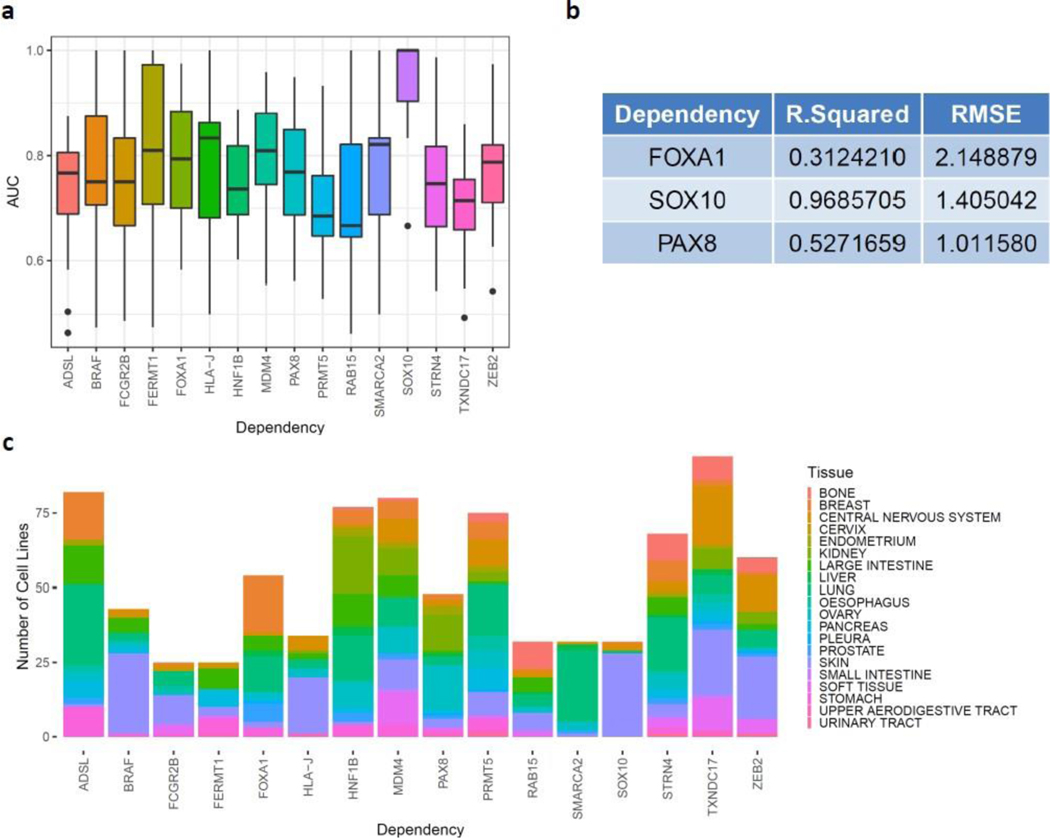
EDDI model performance. (a) Performance of random forest models with respect to 16 identified dosage- based dependencies. Area under curve (AUC) was used as a metric to evaluate classification performance of the selected models; (b) Performance of relaxed-lasso linear regression models of SEESAW-like predictors using R- squared (R2) and root-mean-square-error (RMSE) as performance metrics; (c) Proportions of tissue-type cancer cells that contribute for identified dosage-based dependencies.

To identify dependency with DDP, we used a naïve logistic-regression (Figure 1). Of the 17,098 dependencies, 109 were selected for having possible DDPs. A random forest model was then created for 109 dependencies using the possible pre-screened DDPs to predict dependency and extract feature weights. Using the area under the curve (AUC) as our validation metric (Figure 2a), we found 16 dosage-based dependencies with a cross-validated model performance of greater than 0.7. The predictor genes (i.e. features) are given in Data S1. Out of these 16 dosage- based dependencies, SOX10 dependency was most accurately predicted by these random forest models which is consistent with the previous findings of Tsherniak et al. [2]. We noticed that the variability of predictive performance was not dependent on the number of cancer cell lines (Figure 2a). This indicated that the dissected patterns were driven by true expression patterns and not by under sampling. For instance, STRN4 had the smallest variation in predictive performance, and FERMT1 had the largest variation in predictive performance (Figure 2a). Across cancer types, STRN4 had a robust performance, which is in contrast to SOX10 that had a largely improved performance in skin cancer when compared to other cancer types.

To test the linearity of the 16 highly predictive dosage-based dependencies, we subjected them to relaxed-lasso linear regression models. A leave-one-out cross-validation (LOOCV) was used as a test strategy, and the LOOCV root-mean-square error (RMSE) and R-squared (R2) were used to measure performance. FOXA1, SOX10, and PAX8 displayed significant R2 and RMSE performances (Figure 2b). Interestingly, features selected by this model demonstrated the linear “SEESAW” pattern (Figure S2). The remaining 13 dosage based dependencies were characterize by predictors with over- and under-expression profiles.

### 3.3 Most dosage-based dependencies are related to transcriptional activities

We next examined the functional categories of these 16 dosage-based dependencies. Half of these dependencies were functionally related to transcriptional regulation activities including transcription factors (FOXA1, HNF1B, PAX8, SOX10, ZEB2), transcriptional regulator (MDM4), and chromatin remodeling (PRMT5, SMARCA2). Other functional categories were purine metabolism (ADSL), MAPK signaling (BRAF), calcium ion binding (CABS1, STRN4), immune responses (FCGR2B, HLA-J), integrin signaling and cytoskeleton (FERMT1), and electron transfer activities (TXNDC17). This indicates that dosage-based dependencies in cancer cells are most often related to transcriptional activities. Several of the non-transcriptionally functions associated to dosage-based dependencies were also related to tumorigenesis.

### 3.4 Dosage-based dependencies are tissue specific

After broad characterization, we then analyzed the tissue specificity of dosage-based dependencies. We found that each tissue showed a preference for certain dosage-based dependencies. (Figures 2c and S4). The most obvious example was SOX10 which was dependent in >80% of skin cancer cell lines (Figure 2c). This is consistent with prior studies demonstrating SOX10 as a marker and promotor of invasiveness in melanomas [14, 15]. BRAF was also enriched by skin cancer cell lines (Figure 2c) which was unsurprising because BRAF mutations occur in nearly 70% of cutaneous melanomas [16]. Kidney derived cell lines were dependent on transcription factors HNF1B and PAX8. PAX8 is a known activator of metabolic genes in renal cell carcinoma [17]. HNF1B has not been described in kidney cancer, but dysfunction in HNF1B is known to cause developmental kidney diseases and renal cysts [18, 19].

### 3.5 Dosage dependent predictors were enriched in cancer related molecular pathways

**Figure 3:**
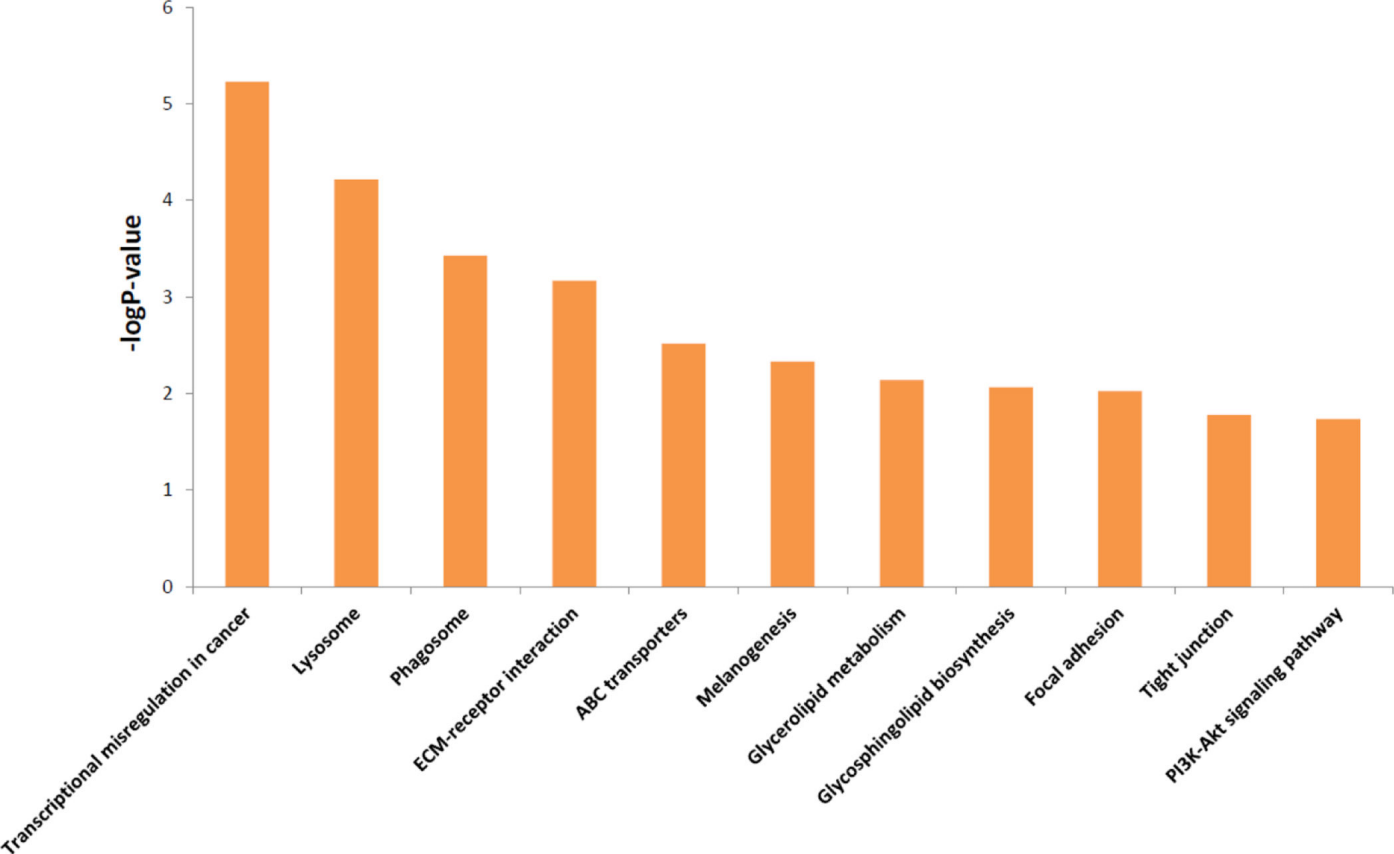
Enriched KEGG pathways of the 16 significant dosage-based dependent predictor genes performed using over-representation analysis from WebGestalt [13].

The 16 gene dependencies were significantly predicted by 1,162 DDPs. Using WebGestalt and the KEGG database, we again found that DDPs were enriched in cancer related pathways (Figure 3 and Data S2) [13, 20]. Transcriptional misregulation was most significantly enriched in DDPs which agrees with the functional analysis of dependencies of which half were related to transcription. Lysosome, phagosome, and ECM-receptor interaction were the next three pathways that DDPs were enriched in. Lysosmal and phagosomal dysregulation is a known aberration in many cancer types, and ECM-receptor interaction changes can promote cancer invasion and metastasis [21, 22]. Other DDP enriched pathways were focal adhesion and tight junctions which are also related to invasion, growth, and metastasis. A substantial number of DDPs were enriched in melanogenesis which is due to the contribution of large number of SOX10’s DDPs and SOX10’s high level of dependency in skin cancers.

### 3.6 Antisense and non-coding RNA transcripts contribute to dosage-based dependencies

**Figure 4:**
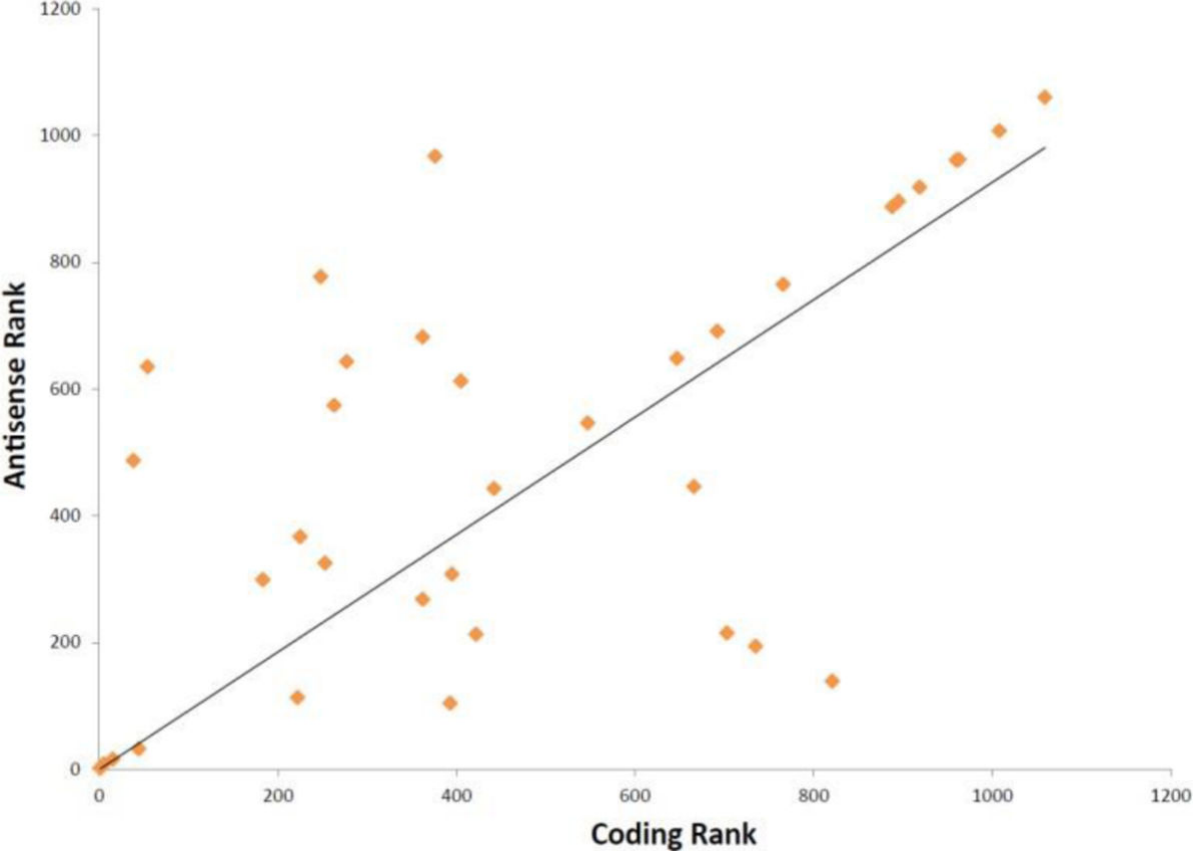
Rank of coding and corresponding anti-sense RNA transcripts in their predictive power using Gini index.

Noncoding RNAs have recently been implicated in cancer therapeutic response and progression, but they have not been described to have a role in gene dependency [23, 24]. Surprisingly, we found 51 antisense transcripts acted as DDPs (Data S1). Not all of the protein-coding counterparts of these transcripts were DDPs which suggests that in the case of gene dependency these antisense RNAs may be playing roles other than regulating the level of their protein-coding counterparts. A number of antisense transcripts whose counterparts were DDPs displayed similar modes of expression (Data S3). We investigated the predictive power of these protein-coding and antisense pairs by comparing the mean decrease in Gini index [25]. A substantial number of antisense transcripts showed similar importance as a model feature to their protein coding analogs (Figure 4). Notably, the antisense transcripts of some genes like GNG12 and PRR7 ranked higher than their protein coding counterparts (Figure 4 and Data S3). This may suggest that under certain circumstances anti-sense RNA transcripts rather than the protein-coding transcripts play a more important role in shaping gene dependencies. Non-coding RNAs were also found to be DDPs in our models (Data S1). We detected 46 non-coding RNAs as DDPs, and some of them appeared as predictors for multiple dependencies. For example, LINC00473 was a DDP for FCGR2B and SOX10 dependencies. Long non-coding RNAs have recently been reported to act as predictors of anticancer drug sensitivity [23], but to our knowledge, no study has yet reported the role of long non-coding RNAs as predictor of gene dependencies in cancer cells.

### 3.7 Distinct modes of expression of dosage-dependent predictors confer unique mechanisms of dependencies

In order to find distinct modes of expression within our DDPs, we combined these results with differential expression analysis. Our model previously found 1,162 DDP-dependency pairs with discernable modes of expression (Data S1 and see Figure S1–S3 for selected examples). The vast majority of DDPs were grouped as over- expression (including separable over expression) and under-expression (separable and suppressed under-expression). These modes of expression were essentially non-linear which are easily identified by the random forest methods used in this study. We then searched for DDPs who exhibit linear relations with dependency scores which we termed “SEESAW” as described above. Only three dependencies, FOXA1, SOX10, and PAX8, had SEESAW expression modes in their respective DDPs (Data S1). SSX1, a transcriptional repressor and potential immunotherapy target [26], was a SEESAW DDPs for SOX10 and PAX8. IL-4I1 (interleukin-4 induced 1), which is involved in catabolism of several aromatic amino acids [27], was a SEESAW-like DDP to PAX8 dependency. Interestingly, we found transcription factors FOXA1 and PAX8 were SEESAW-like DDPs to their own dependencies. These “SEESAW” DDPs represent a distinct mode of action in dependent cell lines, suggesting a unique functional role of the respective gene in dependent cells.

### 3.8 DDP- dependency network analysis revealed the core triad of SOX10, HLA-J, and ZEB2 for dosage based gene dependencies

**Figure 5:**
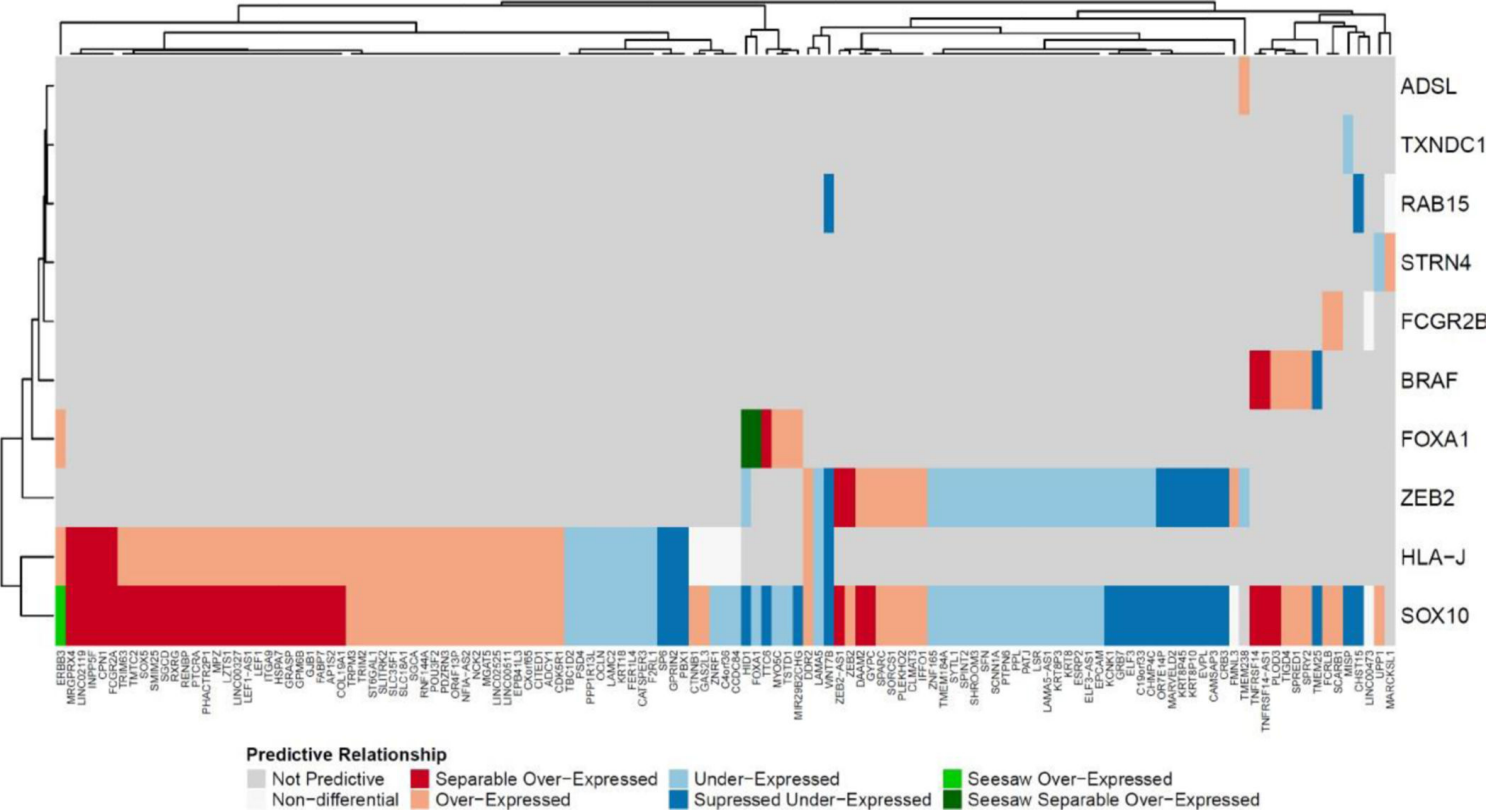
Heatmap of dosage-based dependent predictors (DDPs, bottom) and 10 dependent genes (right) with at least one shared DDPs. Hierarchical clustering was performed to cluster DDPs and dependent genes based on expression modes of DDPs.

Having dependent genes and their respective DDPs relations (Data S1) obtained from our machine learning models, we then construct dependent-predictor networks and utilized the constructed network to explore the degree and mode of shared dependencies. To do this we began by exploring the characteristics of DDPs which were involved in at least two dependencies. SOX10 was predicted by the most genes (962 DDPs). FOXA1, HLA-J, and ZEB2 were also highly populated with DDPs although to much less extent compared to SOX10. The remaining 12 dependencies had only a handful of DDPs, implying dependencies for these genes are modulated by other molecular factors such as genetic mutations and epigenetics. We found the triad of SOX10, HLA-J, and ZEB2 was the core for dosage-based dependencies in our models. Hierarchical clustering on the DDP modes of expression for each dependency revealed that HLA-J and SOX10 were most closely associated with 57 shared DDPs (Figure 5). The shared DDPs between these two dependencies were primarily in the over-expression categories. In contrast, the DDPs shared by SOX10 and ZEB2, 36 in total, were primarily under-expression categories (Figure 5). As shown in Figure 2c, the vast majority of cancer cell lines that contributed to SOX10 dependency were derived from skin cancers. In contrast, HLA-J and ZEB2 dependencies contained substantial dependent cell lines derived from other types of cancer such as pancreas, lung, and liver in addition to a substantial population of skin cells. Cell lines dependent on FOXA1 were derived from multiple types of cancer cells, including breast, large intestine, liver, lung, stomach, and urinary tract suggesting that FOXA1 is a more conserved dependency. These tissues distributions are reflected by the similarity in DDPs between SOX10, ZEB2, and HLA-J and the discrepancy between SOX10 and FOXA1. While FOXA1 shared a number of DDPs with SOX10, the DDP modes were opposite.

**Figure 6:**
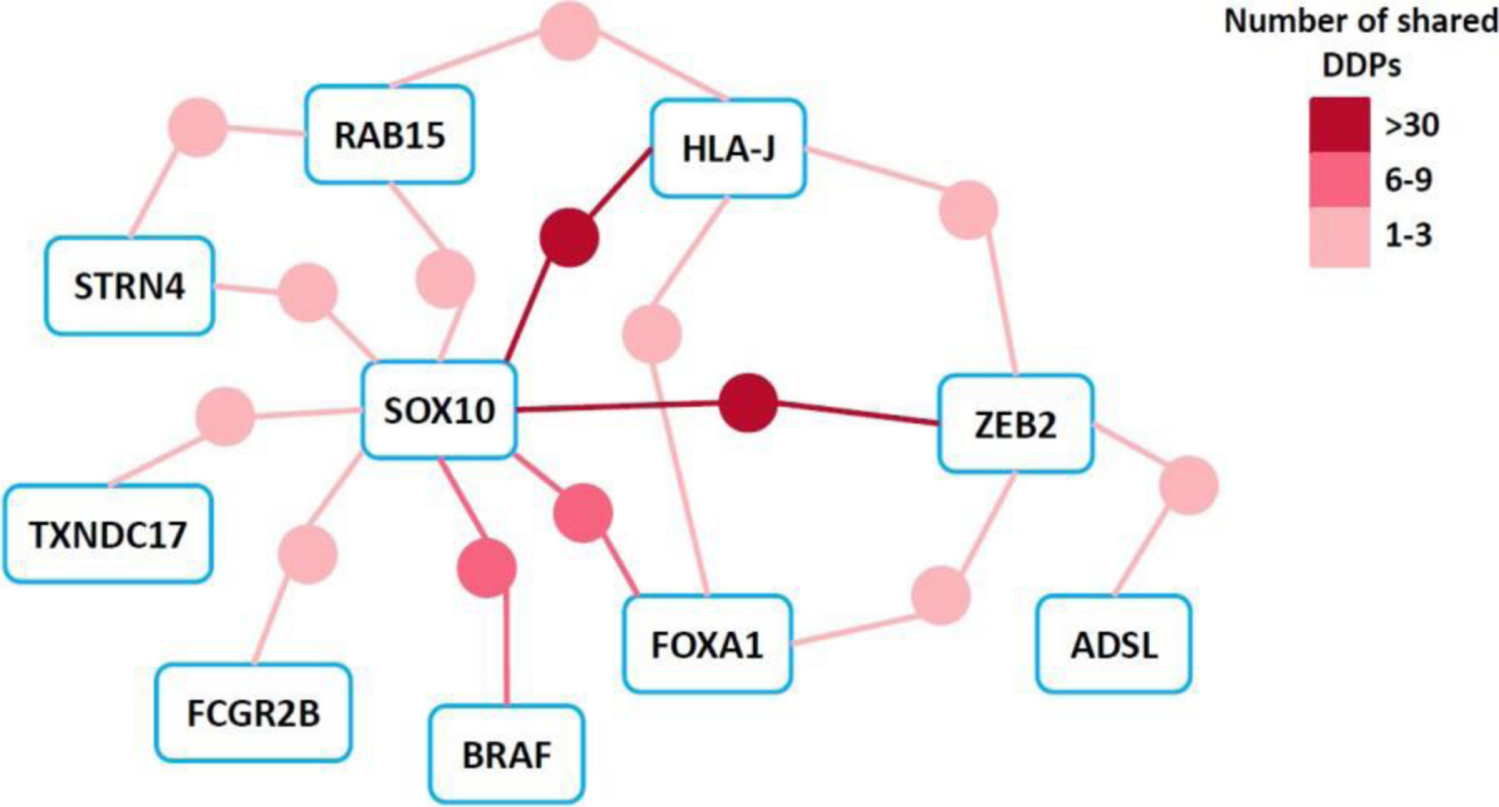
Network of dependent genes (represented as rounded rectangles) connected via shared DDPs (represented as circular nodes, color coded by the number of shared genes). See also Figure S5 for a detailed network representation. DDP: dosage-based dependent predictors.

We then construct dependent-predictor network from these 1,162 pairs to examine the interactions of DDPs between dependencies (Figure 6). SOX10 acted as a hub linking all dependencies but ADSL through shared DDPs. We found that SOX10 interestingly displayed overlapping modes of DDP expression with other dependencies like BRAF, RAB15, ZEB2, HLA-J, and FCGR2B (Figures 5 and 6). FOXA1 interestingly defied this paradigm with two SEESAW and three over-expressed category DDPs that were under-expressed categories in SOX10. Notably, our results suggest connections between SOX10 and BRAF in the etiology of skin cancer and modulating their shared DDPs via chemotherapeutics might open a new avenue to rescue resistance to BRAF/MEK inhibitors in melanoma patients [28].

## 4. Discussion

Gene dependency is a broad umbrella that covers important phenomena like synthetic lethality, gene essentiality, and oncogene addiction [29–31] which are caused by a range of molecular mechanisms, including genetic and epigenetic alterations, change of gene expression, RNA processing, and protein modifications [1–3]. Thus, understanding gene dependency is challenging but nevertheless important for identifying effective targets in therapeutics development. We approached this problem by combining machine learning and systems biology, a combination that has previously revealed expansive new insights in disease biology as evidenced from our previous works [32–35].

Cancer cell lines are excellent models to understand gene dependency because they served as a general platform for omics data generation and screening experiments. Genome-wide screens using RNAi and CRISPR-Cas9 have provided unprecedented opportunities to quantify gene dependency. This screening data can in turn be used to build machine learning-based predictive models where features such as genetic mutations and gene expression levels predict the extent of gene dependency [2, 3, 36]. Such predictions can be additionally used to provide mechanistic insights as we have shown in this study. Previous analysis tackled this problem using a regression task rather than trying to dichotomize into dependent/non-dependent and uncover meaningful biological insights embedded in the machine learning models. We turned this into a classification problem to enhance interpretability. Furthermore, we focused on a specific mode of gene dependency, dosage-based dependency where expression levels of genes were the sole predictive factors.

Our systems biology analysis elucidated biological properties of the 1,162 dependent-predictor pairs identified in our machine learning models. Pathway analysis on dosage-based dependency predictors (DDPs) revealed transcription regulation played key roles in dosage-based dependencies. Interestingly, we also found anti-sense RNAs and long-noncoding RNA transcripts acted as DDPs. To date, no study has reported non-coding RNAs playing a role in determining gene dependency in cancer cells, which suggests that further efforts should be dedicated to understanding non-coding RNAs in gene dependency.

Network analyses revealed SOX10, HLA-J, and ZEB2 was a gene triad that acted as hubs in the dependent-predictor network. Combined analyses of network, mode of expression of DDPs, and cellular context further unveiled the existence of distinct modes of dosage-based dependency for particular genes such as BRAF and FOXA1. In sum, our work shows that it is possible to unearth hidden biological knowledge of gene dependency using trained machine learning models and systems biology.

In summary, we illustrate the feasibility of harnessing the power of both machine learning and systems biology to decipher the biology of cancer gene dependency. We specifically focused on dosage-based gene dependency predictors (DDPs). We found robust evidence of their existence and association with gene dependency. Our results thus open several avenues of therapeutic investigation because we identified up- and downstream factors affecting gene dependency. This work only focuses on dosage-based dependencies and does not explore the full realm of gene dependency which involves other molecular factors such as genetic mutations and epigenetic modifications. Nonetheless, we are convinced that similar approaches will inspire the development of more sophisticated computational models to uncover the biology of gene dependency in the near future.

## Supplementary Material

Supplymentary

Data S1

Data S2

Data S3
