# Supplementary material for: Machine Learning and Systems Biology Approaches to Characterize Dosage-Based Gene Dependencies in Cancer Cells
Source: J Bioinform Syst Biol. Author manuscript; Available in PMC 2021 Apr 8. (PMC8031731)
Supplement: Supplymentary [file NIHMS1678407-supplement-Supplymentary.docx]

## Supplementary Figures


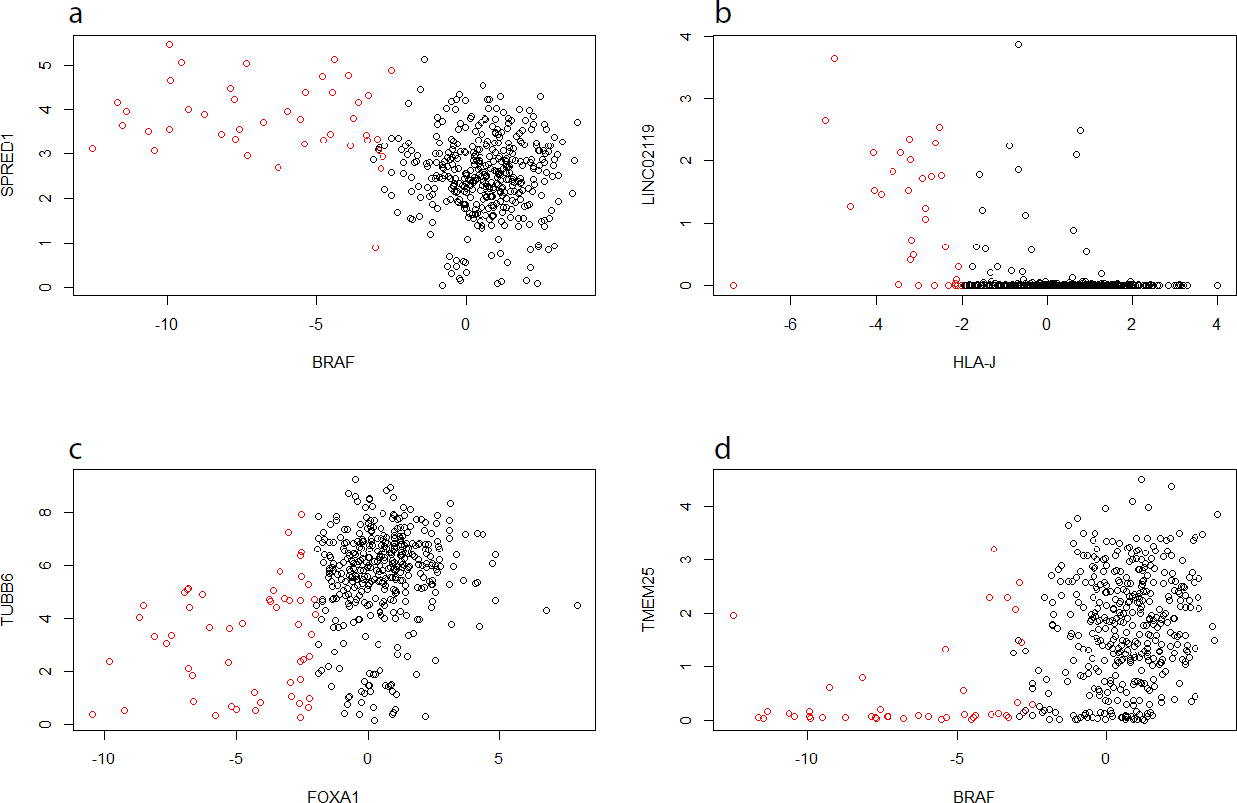


**Figure S1:** Four most common modes of expression of selected DDPs. The gene dependency score is on the horizontal x-axis and the expression of DDP (computed with log2(RPKM+1)) is represented at the vertical y-axis. Individual points are cell lines, with reds denote those cell lines identified as dependent on the corresponding gene and blacks are independent cell lines. (a) Over-expressed; (b) Separable over-expressed; (c) Under-expressed; (d) Suppressed under-expression.


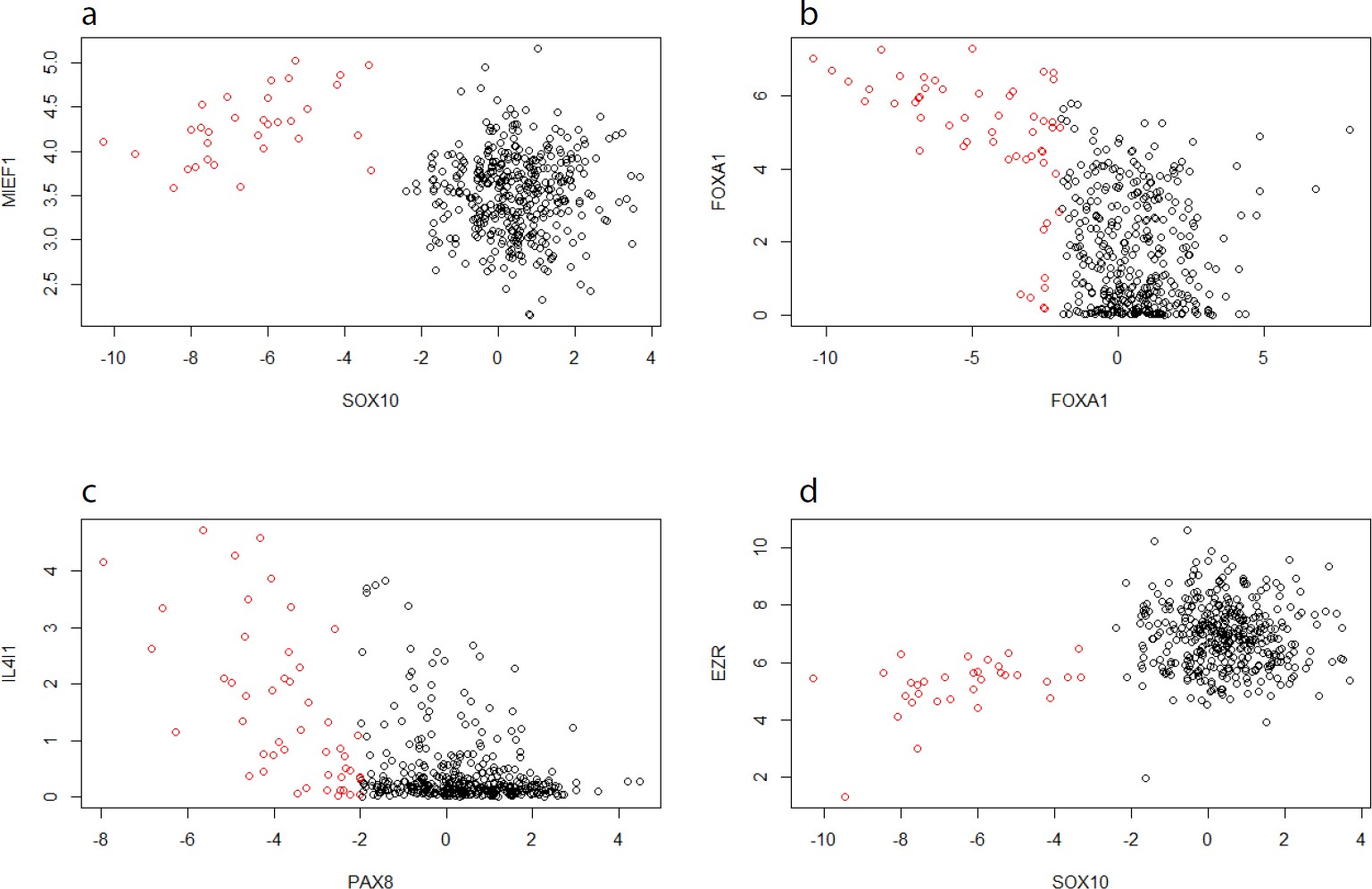


**Figure S2:** SEESAW-like expression modes of selected DDPs. All seesaw patterns demonstrate a clear linear relationship in the dependent cell lines. The gene dependency score is on the horizontal x-axis and the DDP expression computed in log2(RPKM+1) at the vertical y-axis. Individual points are cell lines, with reds denote those cell lines identified as dependent on the corresponding gene and blacks are independent cell lines. (a) Non- differentially expressed SEESAW; (b) Separable over-expression SEESAW; (c) Over-expressed SEESAW; (d) Under-expressed SEESAW.


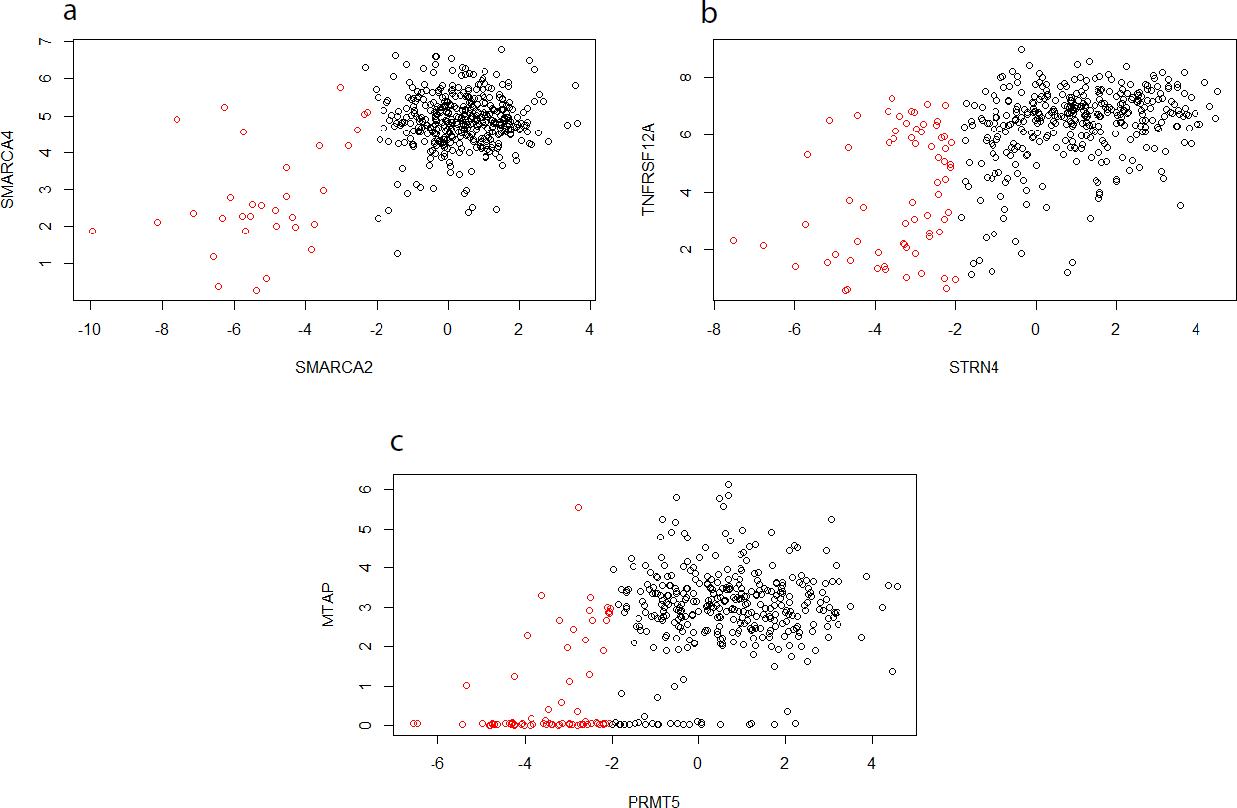


**Figure S3:** Unique under-expression patterns of DDPs. There were three selected DDPs demonstrating unique under-expression patterns. The gene dependency score is on the horizontal x-axis and the DDP expression computed in log2(RPKM+1) at the vertical y-axis. Individual points are cell lines, with reds denote those cell lines identified as dependent on the corresponding gene and blacks are independent cell lines. (a and b) Separable under expression.

(c) Separable, suppressed under-expression.


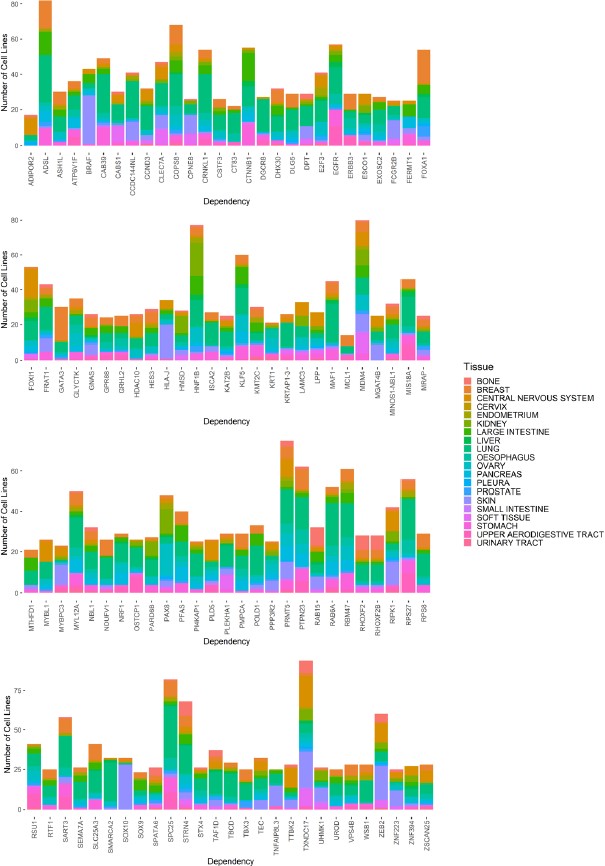


**Figure S4:** Proportion of tissue-type of cancer cell lines present for 109 gene dependencies selected by the initial screen.


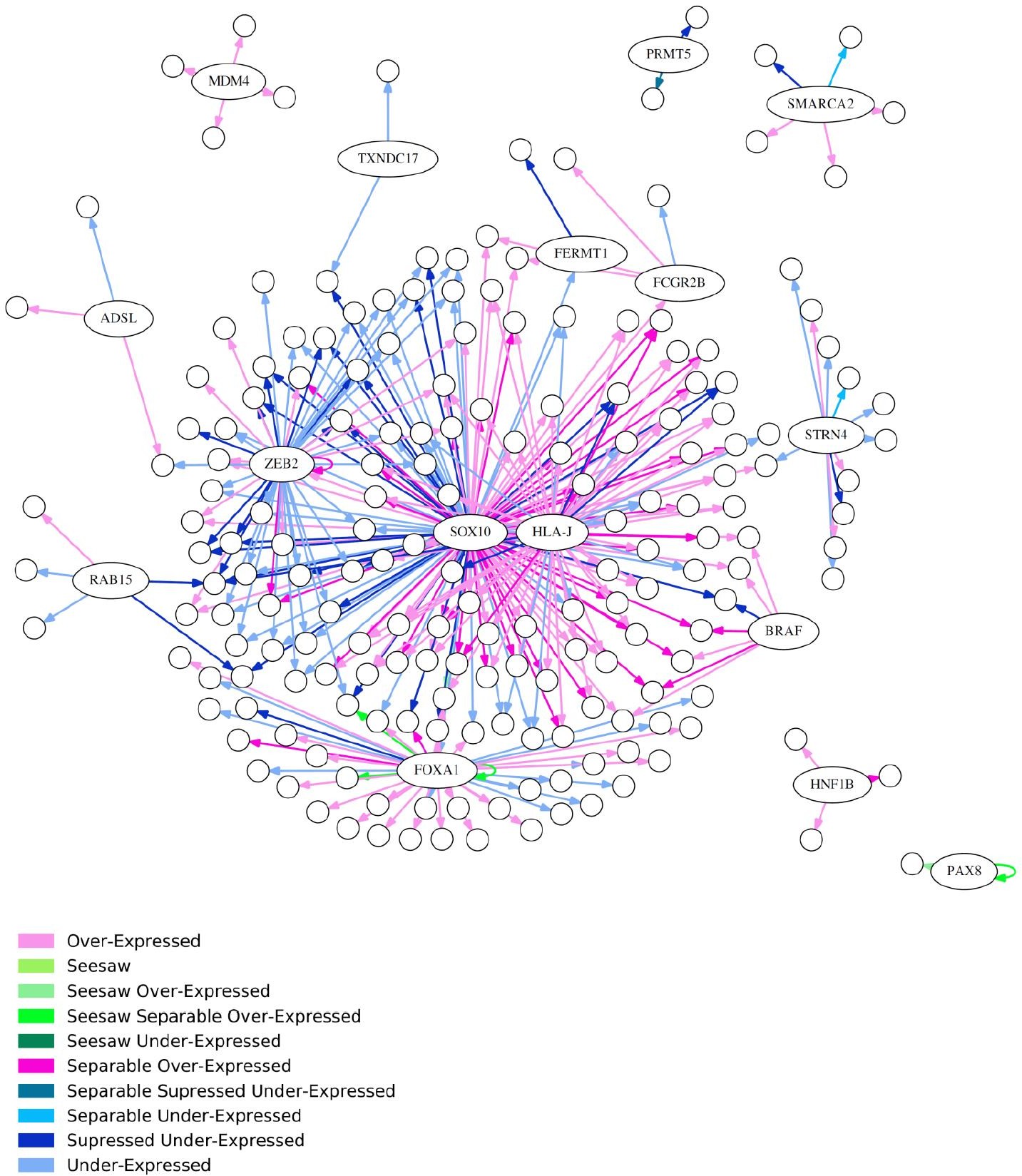


**Figure S5:** Network of 16 dosage-dependent gene dependencies (labeled with gene names in oval nodes) with their respective dosage-based dependent predictors (DDPs, unlabeled circular nodes). To simplify the network, DDPs that connect only to SOX10 are excluded. DDPs modes of expression are represented with corresponding color codes. See also Data S1 for the complete 1162 dependent-predictor pairs.
